# Molecular characterization of a rare case of high-grade B-cell lymphoma with *MYC*, *BCL2*, *BCL6*, and *CCND1* rearrangements

**DOI:** 10.1007/s12308-024-00593-8

**Published:** 2024-06-24

**Authors:** FNU Monika, Ahmed Sabri, David Cantu, Eric Vail, Andrew Siref

**Affiliations:** 1 Department of Pathology, Creighton University, Omaha, NE 68124 USA; 2 Department of Pathology, CHI Health Creighton University, Omaha, NE 68124 USA; 3 Cedars-Sinai Medical Center, Los Angeles, CA 90048 USA

**Keywords:** Quadruple hit lymphoma, *MYC-BCL2* rearrangements, *MYC-BCL6* rearrangements, Lymphadenopathy, *KMT2D*

## Abstract

Quadruple-hit lymphomas are extremely rare non-Hodgkin lymphomas with a reported dismal prognosis in the few reported cases. A “quadruple hit” has been defined by the presence of concurrent *MYC*, *BCL2*, *BCL6*, and *CCND1* chromosomal rearrangements*.* We report a new case of a quadruple hit lymphoma in a 73-year-old Hispanic man who presented with an enlarging left-sided neck mass. Computed tomography showed a 1.9-cm mass in left the tonsil with bulky cervical lymphadenopathy. The presence of all four chromosomal rearrangements can reportedly occur with disease progression in both diffuse large B-cell lymphomas and mantle cell lymphomas. Further characterization of the tumor by next-generation sequencing may be of benefit to delineate between these two possibilities. Immunohistochemistry (IHC), fluorescence in situ hybridization (FISH), and next-generation sequencing were used to confirm and classify the diagnosis. Histologic sections of the cervical lymph node demonstrated an atypical lymphoid infiltrate with large and pleomorphic cells, which were positive for CD20, CD10, BCL1 (Cyclin D1), BCL2, BCL6, and cMYC and negative for CD5 and SOX11 on immunohistochemistry with a Ki-67 proliferative index of 70%. FISH demonstrated *MYC*, *BCL2*, *BCL6*, and *CCND1* rearrangements and the diagnosis of high-grade B-cell lymphoma with *MYC*, *BCL2*, *BCL6*, and *CCND1* was rendered. Our patient was treated with dose adjusted etoposide, doxorubicin, cyclophosphamide, prednisone, and rituximab chemotherapy and has been in remission for 20 months.

**Supplementary Information:**

The online version contains supplementary material available at 10.1007/s12308-024-00593-8.

## Introduction

Diffuse large B-cell lymphoma (DLBCL) is an aggressive and biologically heterogeneous non-Hodgkin lymphoma group of B-cell lymphomas. High-grade B cell lymphoma (HGBL) with translocations involving *MYC* and *BCL2* is retained as a so-called double hit (DH) lymphoma, by the 5th edition World Health Organization (WHO) classification (2022) [1]. DH lymphomas are rare entities with poor prognosis. In the revised 4th edition of the WHO Classification (2017), these lymphomas were categorized as HGBL with *MYC*, *BCL2*, and/or *BCL6* rearrangements. However, the 5th edition WHO classification has excluded *MYC* and *BCL6* rearranged cases from the DH category. B-cell lymphomas with *MYC* and *BCL6* rearrangements are now reclassified as a subtype of DLBCL, NOS or HGBL, NOS according to their cytomorphological features [1]. In comparison, the International Consensus Classification (ICC) has retained these cases as a DH sub-category on the basis that some studies have recorded poor outcomes [2]. “Quadruple-hit” lymphomas, while not a defined entity in either the WHO or ICC classification schema, have been characterized by the concurrent presence of *MYC*, *BCL2*, *BCL6*, and *CCND1* rearrangements, and are extremely rare with an apparent dismal prognosis. Currently, only 10 such cases have been reported in the literature [3–10]. Herein, we report a case of HGBL with *MYC*, *BCL2*, *BCL6*, and *CCND1* rearrangements, a so-called quadruple hit lymphoma and describe its molecular and cytogenetic features.

## Case presentation

A 73-year-old male with no significant past medical history presented with a left-sided neck swelling for a few days’ duration. Initially, he was treated with antibiotics for presumed underlying infection. Three months later, he returned to clinic with further enlargement of swelling in the left neck. Laryngoscopy revealed an enlarged, firm, and erythematous left tonsil with an exophytic mass. The right tonsil was unremarkable. CT scan of the neck demonstrated a 1.9-cm mass in the left tonsil with bulky cervical lymphadenopathy; a subsequent PET scan showed the mass and lymph nodes to be hypermetabolic; no other hypermetabolic lesions were present.

**Fig. 1:**
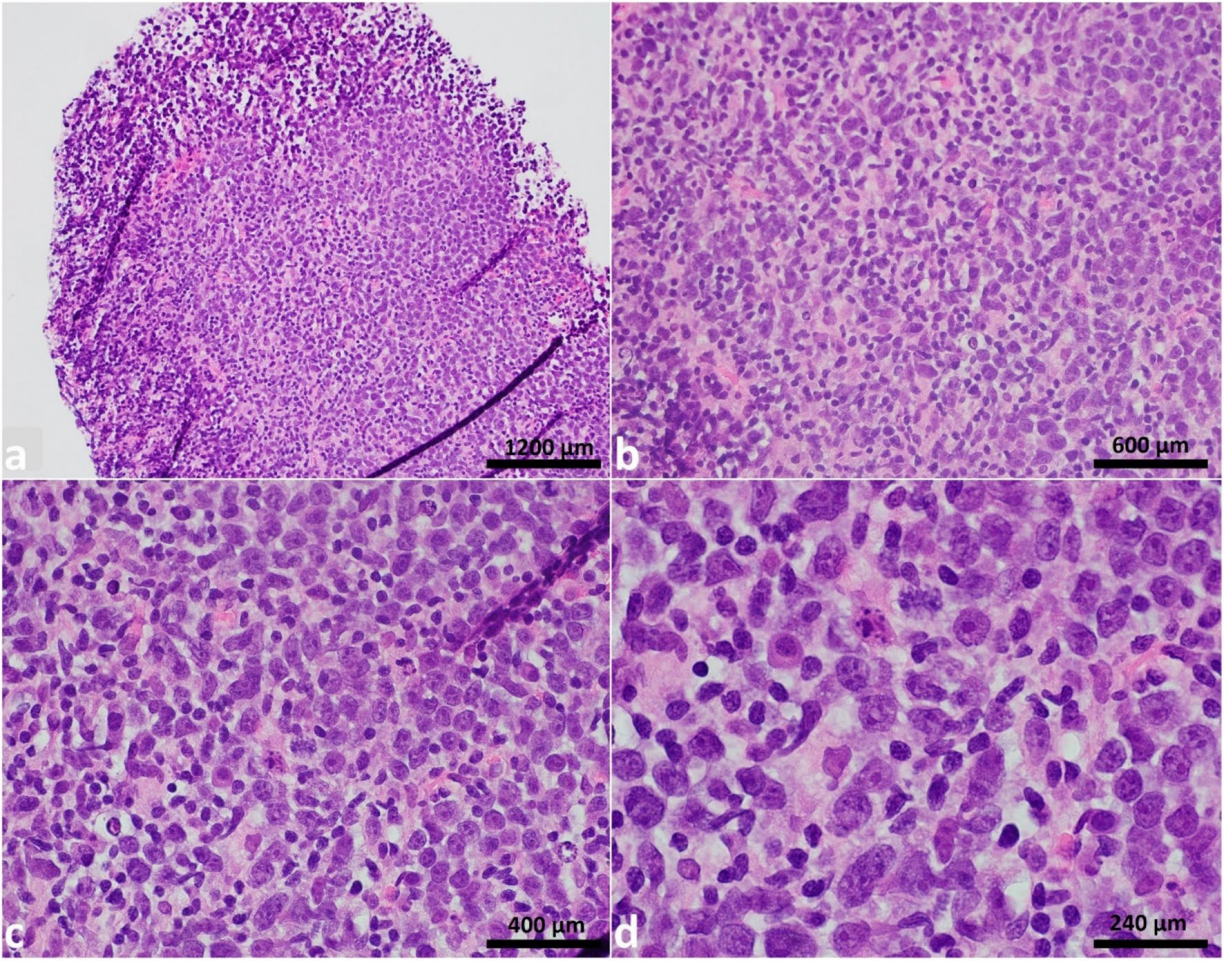
Hematoxylin & eosin (H&E) stain of left cervical lymph node. Complete architectural effacement of lymph node core tissue by large pleomorphic nuclei with irregular contour, blastoid chromatin, and prominent eosinophilic nucleoli. The magnifications in figures **a** to **d** in order are 200 × , 400 × , 600 × , and 1000 ×

**Fig. 2:**
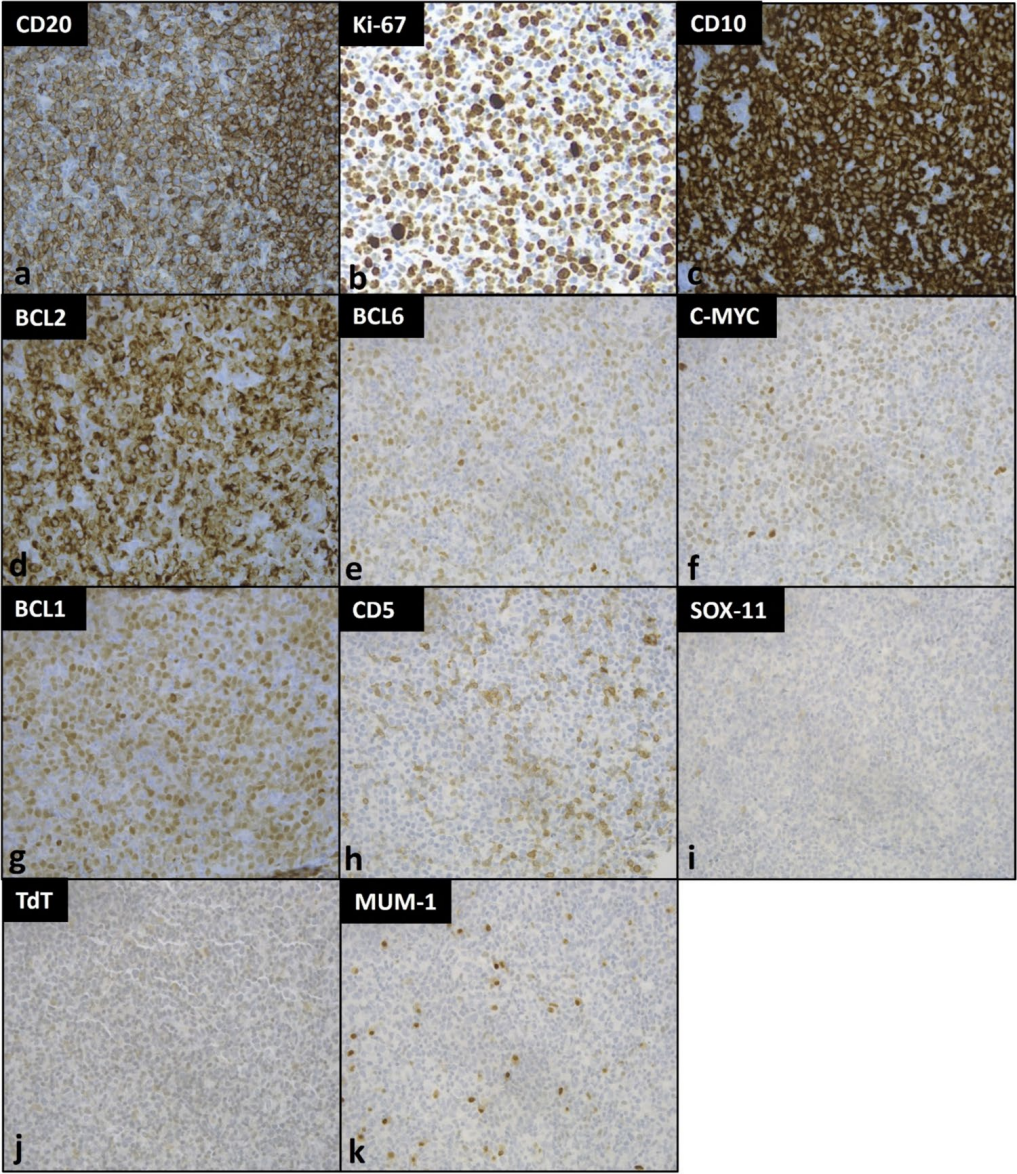
Immunohistochemical stains of left cervical lymph node. The immunohistochemical stains at 400 × magnification show tumor cells positive for **a** CD20, **b** Ki-67, **c** CD10, **d** BCL-2 (> 50%), **e** BCL-6 (weak, > 30%), **f** c-MYC (weak, > 40%), and **g** BCL1, and negative for **h** CD5, **i** SOX-11, **j** TdT, and **k** MUM-1

A core biopsy of the left cervical lymph node was obtained. Histology showed complete architectural effacement by large and pleomorphic cells (Fig. 1) which were positive for CD20, CD10, BCL1, BCL2, BCL6 (subset, weak), and cMYC. The Ki-67 proliferative rate was estimated at 70% (Fig. 2). Tumor cells were negative for CD5, CD23, SOX11, MUM1, TdT, CD30, and EBER by in situ hybridization. Given the overall findings, a preliminary diagnosis of DLBCL of germinal center origin was rendered, pending fluorescence in situ hybridization (FISH) studies to rule out a possible HGBL. In addition to *MYC*, *BCL2*, and *BCL6* probes, testing for *CCND1* rearrangement was also performed due to diffuse expression of BCL1.

**Fig. 3:**
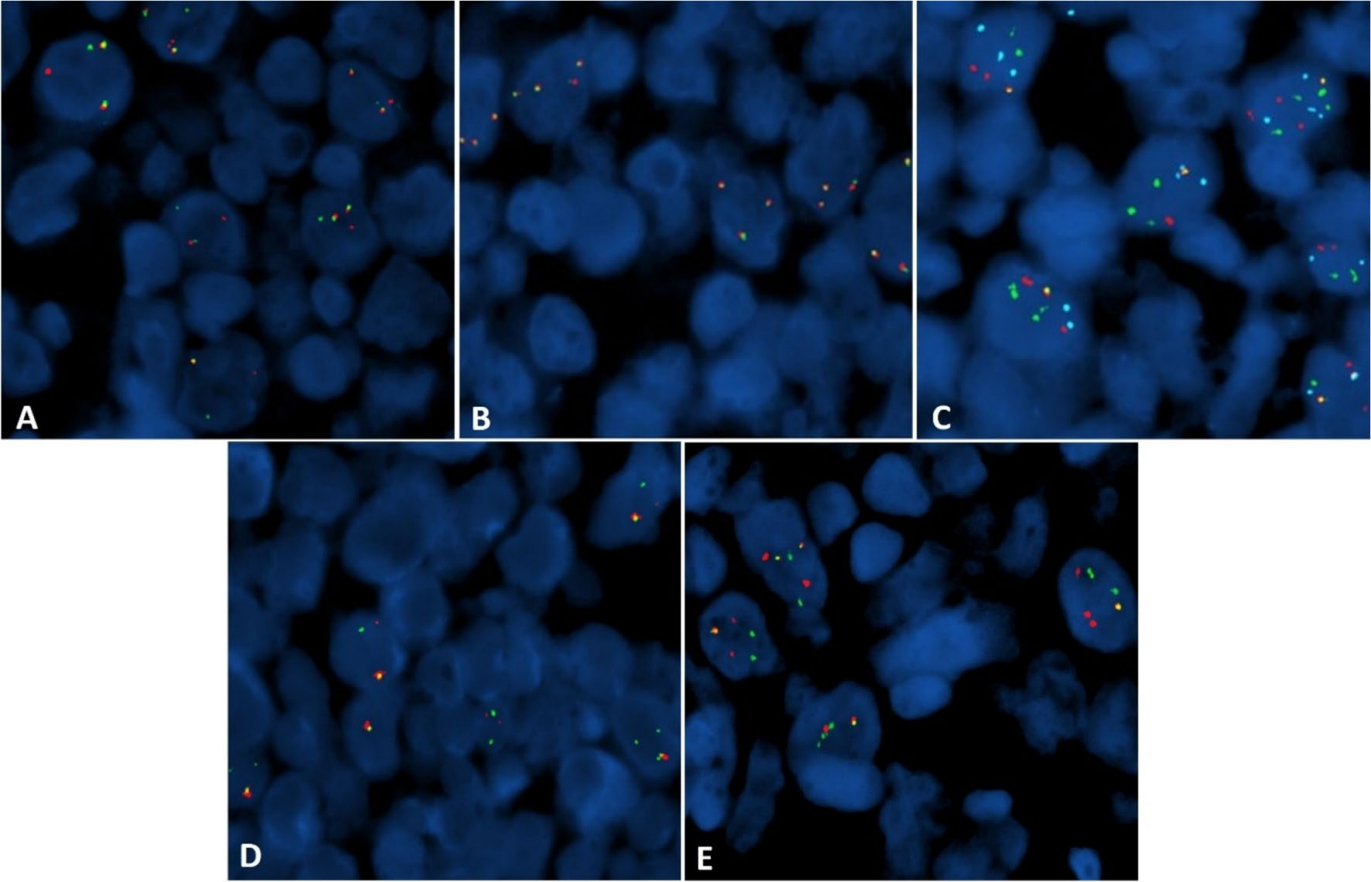
Fluorescence in situ hybridization studies of lymph node (FISH). Break-apart FISH studies showed breaks in *BCL6* (**A**), but no breaks in *MYC* (**B**). Dual fusion FISH studies showed the presence of only a single fusion signal and variable additional probe signals for: *IGH::MYC* {green: *IGH*, red: *MYC* (8q24), blue: CEP8}(**C**), *IGH::BCL2* {green: *IGH*, red: *BCL2*}(**D**), and *IGH::CCND1* {green: *IGH*, red: *CCND1*}(**E**)

FISH studies (Fig. 3) revealed variant *MYC* (84.5%), *CCND1* (77.5%), and *BCL2* (95%) rearrangements, all involving the *IGH* gene locus and showing the presence of only a single fusion signal on dual color FISH. The translocations were specifically identified as t(8;14)(q24;q32), t(11;14)(q13;q32), and t(14;18)(q32;q21), respectively. Rearrangement of *BCL6* (3q27) (72.5%) was also detected on break-apart probe testing. Studies were initially negative for rearrangement of the *MYC* (8q24) locus using a dual color break-apart probe; however, 3–4 intact (non-rearranged) copies of 8q24 were observed in 55% of the cells analyzed, prompting the *MYC*::*IGH* fusion testing with dual fusion probes. FISH studies also demonstrated the presence of trisomy 8 and additional signals for *IGH*, *BCL6*, and *CCND1*. No fusion partner testing was performed for *BCL6*, or for potential partners from the variant rearrangements of *MYC*, *BCL2*, and *CCND1*.

Based on the FISH results, a modified diagnosis of HGBL with *MYC*, *BCL2*, *BCL6*, and *CCND1* rearrangements was made. Next-generation-sequencing (NGS) was performed for further characterization, with results detailed in Table 1. No abnormalities were seen in the *TP53* gene. Tumor mutation burden is calculated as 7.55 mutations/Mb.

**Table 1:** Gene mutations in the present case

Gene name	Chr	Exon (E)/intron (I)	Nucleotide	Amino acid change	VAF	Mutation type	Variant classification
*ARID1A*	1	E: 1/20	c.837_862del	p.Ser280AlafsTer111	11%	Frameshift variant	Likely pathogenic
*BCL2*	18	I: 2/2	c.585 + 4G > C	-	5%	Splice region variant and intron variant	Uncertain significance
*CCND3*	6	E: 5/5	c.774_775delinsTG	p.Ser259Ala	42%	Missense variant	Uncertain significance
*FANCA*	16	E: 31/43	c.3032G > A	p.Arg1011His	7%	Missense variant	Uncertain significance
*KMT2D*	12	E: 32/54	c.8200C > T	p.Arg2734Ter	43%	Stop gained	Pathogenic
*MYC*	8	E: 2/3	c.475C > T	p.Leu159Phe	14%	Missense variant	Uncertain significance
*MYC*	8	E: 2/3	c.339G > C	p.Gln113His	13%	Missense variant	Uncertain significance
*MYC*	8	E: 2/3	c.265 T > A	p.Tyr89Asn	12%	Missense variant	Uncertain significance
*PIM1*	6	E: 2/6	c.113A > T	p.Tyr38Phe	9%	Missense variant	Uncertain significance
*PIM1*	6	E: 2/6	c.87G > C	p.Lys29Asn	8%	Missense variant	Uncertain significance
*PIM1*	6	E: 1/6	c.68C > T	p.Thr23Ile	7%	Missense variant	Uncertain significance
*PIM1*	6	E: 4/6	c.286G > C	p.Val96Leu	7%	Missense variant	Uncertain significance
*PXDNL*	8	E: 11/23	c.1357A > T	p.Thr453Ser	8%	Missense variant	Uncertain significance
*SF3B1*	2	E: 14/25	c.1998G > C	p.Lys666Asn	9%	Missense variant	Likely pathogenic
*SOCS1*	16	E: 2/2	c.46G > A	p.Ala16Thr	7%	Missense variant	Uncertain significance
*TNFRSF14*	1	E: 5/8	c.472C > T	p.Gln158Ter	8%	Stop gained	Likely pathogenic
*TSC2*	16	E: 2/42	c.58G > T	p.Gly20Ter	8%	Stop gained	Likely pathogenic

*Chro* chromosome, *VAF* variant allele frequency

A staging bone marrow biopsy was negative for lymphoma (clinical stage I). The patient was treated with 6 cycles of dose adjusted etoposide, doxorubicin, cyclophosphamide, prednisone, and rituximab (DA-R-EPOCH). This regimen was completed and restaging PET scan at 2 months showed complete resolution of the hypermetabolic lesions. He has been in complete remission for approximately 20 months.

## Material and methods

### Immunohistochemistry

IHC was performed per routine hospital procedures at CHI Health Bergan Mercy Hospital, Omaha, NE, according to the manufacturer’s protocols.

### Fluorescence in situ hybridization

FISH was performed at the Warren G. Sanger Human Genetics Laboratory at Nebraska Medicine, Omaha, NE, utilizing the Locus Specific Identifier (LSI) *IGH*::*MYC* t(8;14) Dual Fusion Translocation Probe with CEP 8, the LSI *IGH*::*CCND1* t(11;14) Dual Fusion Translocation Probe, the LSI *IGH*::*BCL2* t(14;18) Dual Fusion Translocation Probe, the LSI *BCL6* (3q27) Major and Alternate Breakpoint Dual Color Break-apart Probe, and the LSI *MYC* (8q24) Dual Color Break-apart Probe. Standard FISH protocol (co-denaturation of the probe and target at 74 °C for 4 min, hybridization overnight at 37 °C, washing at 72 °C for 2 min) was followed and images were captured using ASI software (Applied Spectral Imaging, Chicago, IL).

### Molecular pathology

NGS was performed at Cedars-Sinai Medical Center, Los Angeles, CA, with further details provided in supplementary material.

## Discussion

DLBCL is an aggressive and heterogeneous grouping of non-Hodgkin lymphomas encompassing a spectrum of different immunophenotypic and molecular variants. The definition and categorization of DLBCL with multiple gene rearrangements (so-called hits) have been evolving in recent years. The 2022-ICC retains a subgrouping for cases with *MYC* and *BCL6* rearrangement; this is recognized as a heterogeneous category with variable gene expression profiles and mutational spectra [2]. Neither the WHO nor ICC classification schema recognize HGBL with *MYC*, *BCL2*, *BCL6*, and *CCND1* rearrangements (a so-called quadruple hit) as a unique entity.

The tumor cells in our case showed mild pleomorphism with eosinophilic nucleoli and some blastoid chromatin. Tumor cells were positive for BCL1, raising the possibility of a blastoid/pleomorphic variant of mantle cell lymphoma (MCL). However, CD5 and SOX11 were negative, arguing against a diagnosis of MCL. TdT was also negative. Tumor cells were also positive for CD10 and BCL6 (subset), further supporting germinal center derivation. Following the currently accepted classification schema, our case is best classified as a HGBL with *MYC* and *BCL2* rearrangement.

B-cell lymphomas with *MYC*, *BCL2*, *BCL6*, and *CCND1* rearrangements are rare entities. Available outcomes data from previous publications suggest this entity has a poor prognosis [3–10]. Among the reported cases, there were four males and three females with median age 74 years (range 51–81 years); lymphadenopathy was seen in four of the seven cases. Two cases reported staging information, and both were stage III. Most cases were negative for CD5 and SOX11 with Ki-67 in the range of 60–90%. Demographic data was not provided for the three remaining cases. There is no consensus on the optimal treatment and the outcomes have been dismal, despite aggressive initial therapies [7]. The clinical features of all reported cases are summarized in Supplementary Table 1 and the histopathological features, diagnosis, ancillary studies, treatment, and outcome/overall survival are summarized in Supplementary Table 2.

*CCND1* gene rearrangement or other genetic alterations involving the gene can lead to aberrant BCL1 protein expression. Among hematolymphoid tumors, BCL1 is found to be overexpressed in > 90% of MCL cases and about 40% of plasma cell myelomas, both of which are caused by a translocation which juxtaposes the immunoglobulin heavy chain (*IGH*) gene to the *CCND1* gene. BCL1 protein expression is rarely seen in DLBCL, which has been linked to copy number gains of *CCND1* or via mRNA dysregulation [11, 12]. FISH studies in our case demonstrated additional signals of *CCND1*, a likely mechanism for the observed protein expression by IHC.

In DLBCL, the data suggests that the *CCND1* rearrangement is a secondary event during lymphoma evolution [12]. Cheng et al. included a quadruple hit lymphoma in their report of DLBCL with *CCND1* rearrangements considered to represent secondary genetic events [9]. This is in contrast to MCL, where *CCND1* rearrangement is considered to be a primary genetic event [12]. MCL may also gain secondary *BCL2*, *BCL6*, and *MYC* rearrangements, as proposed in three [3, 9, 10] of the previously reported quadruple hit lymphomas.

In our case, it is difficult to confidently determine the sequence of genetic alterations. All translocations were found in relatively (and similarly) high proportions of tumor cells. However, sequencing results were more typical of DLBCL than MCL. The combination of mutations best fits in the EZB-DLBCL molecular cluster according to Morin et al. [13]; similar reported molecular clusters include C3, BCL2, and *MYC/BCL2*-DH [14]. Interestingly, a previously sequenced quadruple hit lymphoma, designated as a pleomorphic MCL, shows limited overlapping mutations with our case [10]. The *PIM1* gene was the sole shared mutation with our case. Mutations in the *PIM1* gene are frequently seen in lymphomas and have been implicated in DLBCL pathogenesis [15]. Although *PIM1* mutations identified in our case are variants of uncertain significance (VUS), three out of the four mutations (c.87G > C, c.68C > T, and c.286G > C) have been reported multiple times in DLBCL [16–18] respectively; the other *PIM1* mutation (c.113A > T) has not been reported to our knowledge. NGS findings from the present case and the two previously sequenced quadruple hit lymphomas are summarized in Supplementary Table 3.

*MYC* mutations in DLBCL are more frequently seen in cases with *MYC* and *BCL6* rearrangement, which was present in our case [19]. This is thought to be due to aberrant somatic hypermutation from activation-induced cytidine deaminase, also implicated in the genesis of *MYC* rearrangement [20]. All three *MYC* mutations in our case are identified as VUS and have not been previously reported. Cases exhibiting intact (non-rearranged) copies of *MYC* on break apart probe testing, including some cases with additional intact *MYC* signals, as in our case, have revealed the presence of *MYC* gene fusions when followed by an *IGH::MYC* dual fusion probe [21].

FISH studies also showed additional copies of *BCL6*. This finding has been seen in nearly half of *MYC*-rearranged DLBCLs according to one study [22].

*KMT2D* is frequently mutated in DLBCL (~ 30% of de novo cases, including both germinal center B-cell and activated B-cell subtypes and follicular lymphoma (~ 90%)) [23]. Recent studies have suggested *KMT2D* mutations represent early events in a common progenitor before divergent evolution of follicular lymphoma or DLBCL, the latter occurs through acquisition of additional genetic lesions and clonal expansion [23, 24]

Among lymphomas, *TNFRSF14* mutation has been reported in follicular lymphoma as well as DLBCL, NOS (EZB) [25] *TNFRSF14* mutations have not been reported in mantle cell lymphoma [9]. The specific *TNFRSF14* mutation found in our case (c.472C > T) has been previously reported in follicular lymphoma and DLBCL [26].

In addition to *BCL2* translocation, our case also harbored *BCL2* gene mutation (c.585 + 4G > C). This was categorized as a VUS for our case but been previously reported in follicular lymphoma and DLBCL [27].

B-cell lymphomas with concurrent *MYC*, *BCL2*, *BCL6*, and *CCND1* rearrangements appear to be a rare occurrence; however, current standard approaches to DLBCL/HGBL classification do not require routine testing for *CCND1* rearrangement. With the current classification schemes de-emphasizing the importance of *BCL6* rearrangement, this may no longer be routinely assessed as well. The addition of *CCND1* rearrangement in the workup for a DLBCL/HGBL might only be sought in cases with BCL1 protein expression, as seen in our case. Sequencing may be of benefit for delineating DLBCL from MCL in the quadruple hit setting, although current data is limited.

### Supplementary Information

Below is the link to the electronic supplementary material.

Supplementary file1 (DOCX 103 KB)
