# Supplementary material for: Molecular characterization of a rare case of high-grade B-cell lymphoma with MYC, BCL2, BCL6, and CCND1 rearrangements
Source: J Hematop. 2024 Jun 24;17(3):155–61. doi: 10.1007/s12308-024-00593-8 (PMC11324669; doi:10.1007/s12308-024-00593-8)
Supplement: Supplementary file 1 — Supplementary file1 (DOCX 103 KB) [file 12308_2024_593_MOESM1_ESM.docx]

**SUPPLEMENTARY MATERIAL**

**METHODS**

**Immunohistochemistry**

Paraffin-embedded sections from the lymph node biopsy were stained using antibodies against CD20, CD10, BCL2, BCL6, BCL1, MUM1/IRF4, CD3, CD5, CD21, CD23, SOX11, cMYC, CD34, TdT, CD30, p40, p16, AE1/3 and Ki-67. The stain interpretations for CD10, BCL2, BCL6, MUM1, cMYC, and Ki-67 was assessed semi-quantitatively for the evaluation of cell of origin and double expresser phenotype. EBV testing was performed by EBER ISH.

**Cytogenetics**

FISH studies were performed utilizing the Locus Specific Identifier *IGH::MYC* *t(8;14)* Dual Fusion Translocation Probe with CEP 8, the LSI *IGH::CCND1 t(11;14)* Dual Fusion Translocation Probe, the LSI *IGH::BCL2* *t(14;18)* Dual Fusion Translocation Probe, the LSI *BCL6* *(3q27)* Major and Alternate Breakpoint Dual Color Break-apart Probe and the LSI *MYC (8q24)* Dual Color Break-apart Probe. For reference, the abnormal range for *IGH::MYC*, *IGH::CCND1*, or *IGH::BCL2* fusion is 6-100%, beak-apart rearrangement of *BCL6* or *MYC* is 15-100%, and for multiple chromosome copies it is 10-100%. FISH study was performed on 1200 interphase cells using 9 probes in 5 hybridization areas. Standard FISH protocol (co-denaturation of the probe and target at 74°C for 4 min, hybridization overnight at 37°C, washing at 72°C for 2 min) was followed and images were captured using ASI software (Applied Spectral Imaging, Chicago, IL).

**Molecular Pathology**

Genomic DNA and RNA were extracted from macro-dissected areas on formalin-fixed paraffin-embedded tissue sections.  Concurrent DNA and RNA libraries were constructed using Oncomine^TM^ Comprehensive Plus Assay library preparation kits (ThermoFisher Scientific, Waltham, MA) and the library pools were sequenced using the Ion GeneStudio S5 Prime system (ThermoFisher Scientific). The sequencing data was analyzed by Ion Reporter Software v5.18 and in house developed bioinformatics pipelines. The assay detects SNVs, small indels, CNVs and select fusions from over 500 genes and accurately assesses tumor mutation burden (TMB).

The genes covered are given below:

**SNVs and Indels***: ABL1, ABL2, ABRAXAS1, ACVR1, ACVR1B, ACVR2A, ADAMTS12, ADAMTS2, AKT1, AKT2, AKT3, ALK, AMER1, APC, AR, ARAF, ARHGAP35, ARID1A, ARID1B, ARID2, ARID5B, ASXL1, ASXL2, ATM, ATP1A1, ATR, ATRX, AURKA, AURKC, AXIN1, AXIN2, AXL, B2M, BAP1, BARD1, BCL2, BCL2L12, BCL6, BCOR, BCR, BLM, BMP5, BMPR2, BRAF,  BRCA1, BRCA2, BRIP1, BTK, CACNA1D, CALR, CARD11, CASP8, CBFB, CBL, CCND1, CCND2, CCND3, CCNE1, CD79B, CD274, CD276, CDC73, CDH1, CDH4, CHD10, CDK4, CDK6, CDK12, CDKN1A, CDKN1B, CDKN2A, CDKN2B, CDKN2C, CHEK1, CHEK2, CIC, CIITA, CREBBP, CSF1R, CSMD3, CTCF, CTLA4, CTNNB1, CUL1, CUL3, CUL4A, CUL4B, CYLD, CYP2C9, CYP2D6, CYSLTR2, DAXX, DDR2, DDX3X, DGCR8, DICER1, DNMT3A, DOCK3, DPYD, DROSHA, DSC1, DSC3, E2F1, EGFR, EIF1AX, ELF3, ENO1, EP300, EPAS1, EPCAM, EPHA2, ERAP1, ERAP2, ERBB2, ERBB3, ERBB4, ERCC2, ERCC4, ERCC5, ERRFI1, ESR1, ETV6, EZH2, FAM135B, FANCA, FANCC, FANCD2, FANCE, FANCF, FANCG, FANCI, FANCL, FANCM, FAS, FAT1, FBXW7, FGF7, FGFR1, FGFR2, FGFR3, FGFR4, FLT3 FLT4 FOXA1, FOXL2, FOXO1, FUBP1, GATA2, GATA3, GLI1, GNA11, GNA13, GNAQ, GNAS, GPS2, H2BC5 (HIST1H2BD), H3-3A (H3F3A), H3-3B (H3F3B),H3C2 (HIST1H3B), HDAC2, HDAC9, HIF1A, HLA-A, HLA-B, HNF1A, HRAS, ID3, IDH1, IDH2, IKBKB, IL6ST, IL7R, INPP4B, IRF4, IRS4, JAK1, JAK2, JAK3, KDM5C, KDM6A, KDR, KEAP1, KIT, KLF4, KLF5, KLHL13, KMT2A, KMT2B, KMT2C, KMT2D, KNSTRN, KRAS, LARP4B, LATS1, LATS2, MAGOH, MAP2K1, MAP2K2, MAP2K4, MAP2K7, MAP3K1, MAP3K4, MAPK1, MAPK8, MAX, MDM4, MECOM, MED12, MEF2B, MEN1, MET, MGA, MITF, MLH1, MLH3, MPL, MRE11, MSH2, MSH3, MSH6, MTAP, MTOR, MTUS2, MUTYH, MYC, MYCN, MYD88, MYOD1, NBN, NCOR1, NF1, NF2, NFE2L2, NOTCH1, NOTCH2, NOTCH3, NOTCH4, NRAS, NSD2, NT5C2, NTRK1, NTRK2, NTRK3, NUP93, PALB2, PARP1, PARP2, PARP3, PARP4, PAX5, PBRM1, PCBP1, PDCD1, PDCD1LG2, PDGFRA, PDGFRB, PDIA3, PGD, PHF6, PIK3C2B, PIK3CA, PIK3CB, PIK3CD, PIK3CG, PIK3R1, PIK3R2, PIM1, PLCG1, PMS1, PMS2, POLD1, POLE, POT1, PPM1D, PPP2R1A, PPP2R2A, PPP6C, PRDM1, PRDM9, PRKACA, PRKAR1A, PSMB8, PSMB9, PSMB10, PTCH1, PTEN, PTPN11, PTPRD, PTPRT, PXDNL, RAC1, RAD50, RAD51, RAD51B, RAD51C, RAD51D, RAD52, RAD54L, RAF1, RARA, RASA1, RASA2, RB1, RBM10, RECQL4, RET, RGS7, RHEB, RHOA, RICTOR, RIT1, RNASEH2A, RNASEH2B, RNASEH2C, RNF43, ROS1, RPA1, RPL5, RPL10, RPL22, RUNX1, RUNX1T1, SDHA, SDHB, SDHC, SDHD, SETBP1, SETD2, SF3B1, SIX1, SIX2, SLCO1B3, SMAD2, SMAD4, SMARCA4, SMARCB1, SMC1A, SMO, SNCAIP, SOCS1, SOS1, SOX2, SOX9, SPEN, SPOP, SRC, SRSF2, STAG2, STAT1, STAT3, STAT5B, STAT6, STK11, SUFU, TAF1, TAP1, TAP2, TBX3, TCF7L2, TERT, TET2, TGFBR1, TGFBR2, TMEM132D, TNFAIP3, TNFRSF14, TOP1, TP53, TP63, TPMT, TPP2, TRRAP, TSC1, TSC2, TSHR, U2AF1, UGT1A1, USP8, USP9X, VHL, WAS, WT1, XPO1, XRCC2, XRCC3, ZBTB20, ZFHX3, ZMYM3, ZNF217, ZNF429, ZRSR2***CNVs***: ABCB1, ABL1, ABL2, ABRAXAS1, ACVR1B, ACVR2A, ADAMTS12, ADAMTS2, AKT1, AKT2, AKT3, ALK, AMER1, APC, AR, ARAF, ARHGAP35, ARID1A, ARID1B, ARID2, ARID5B, ASXL1, ASXL2, ATM, ATR, ATRX, AURKA, AURK, AXIN1, AXIN2, B2M, BAP1, BARD1, BCL2, BCL2L12, BCL6, BCOR, BLM, BMPR2, BRAF, BRCA1, BRCA2, BRIP1, CARD11, CASP8, CBFB, CBL, CCND1, CCND2, CCND3, CCNE1, CD274, CD276, CDC73, CDH1, CDH10, CDK4, CDK6, CDK12, CDKN1A, CDKN1B, CDKN2A, CDKN2B, CDKN2C, CHD4, CHEK1, CHEK2, CIC, CREBBP, CSMD3, CTCF, CTLA4, CTNND2, CUL3, CUL4A, CUL4B, CYLD, CYP2C9, DAXX, DDR1, DDR2, DDX3X, DICER1, DNMT3A, DOCK3, DPYD, DSC1, DSC3, EGFR, EIF1AX, ELF3, EMSY, ENO1, EP300, EPCAM, EPHA2, ERAP1, ERAP2, ERBB2, ERBB3,ERBB4, ERCC2, ERCC4, ERRFI1, ESR1, ETV6, EZH2, FAM135B, FANCA, FANCC, FANCD2, FANCE, FANCF, FANCG, FANCI, FANCL, FANCM, FAT1, FBXW7, FGF19, FGF23, FGF3,FGF4, FGF9, FGFR1, FGFR2, FGFR3, FGFR4, FLT3, FLT4, FOXA1, FUBP1, GATA2, GATA3, GNA13, GNAS, GLI3, GPS2, H3-3A (H3F3A), H3-3B (H3F3B), HDAC2, HDAC9, HLA-A, HLA-B, HNF1A, IDH2, IGF1R, IKBKB, IL7R, INPP4B, JAK1, JAK2, JAK3, KDM5C, KDM6A, KDR, KEAP1, KIT, KLF5, KMT2A, KMT2B, KMT2C, KMT2D, KRAS, LARP4B, LATS1, LATS2, MAGOH, MAP2K1, MAP2K4, MAP2K7, MAP3K1, MAP3K4, MAPK1, MAPK8, MAX, MCL1, MDM2, MDM4, MECOM, MEF2B, MEN1, MET, MGA, MITF, MLH1, MLH3, MPL, MRE11, MSH2, MSH3, MSH6, MTAP, MTOR, MUTYH, MYC, MYCL, MYCN, MYD88, NBN, NCOR1, NF1, NF2, NFE2L2, NOTCH1, NOTCH2, NOTCH3, NOTCH4, NRAS, NTRK1, NTRK3, PALB2, PARP1, PARP2, PARP3, PARP4, PBRM1, PCBP1, PDCD1, PDCD1LG2, PDGFRA, PDGFRB, PDIA3, PGD, PHF6, PIK3CA, PIK3CB, PIK3R1, PIK3R2, PIM1, PLCG1, PMS1,  PMS2, POLD1, POLE, POT1, PPM1D, PPP2R1A, PPP2R2A, PPP6C, PRDM1, PRDM9,  PRKACA, PRKAR1A, PTCH1, PTEN, PTPN11, PTPRT, PXDNL, RAC1, RAD50, RAD51, RAD51B, RAD51C, RAD51D, RAD52, RAD54L, RAF1, RARA, RASA1, RASA2, RB1, RBM10, RECQL4, RET, RHEB, RICTOR, RIT1, RNASEH2A, RNASEH2B, RNF43, ROS1, RPA1, RPS6KB1, RPTOR, RUNX1, SDHA, SDHB, SDHD, SETBP1, SETD2, SF3B1, SLCO1B3, SLX4, SMAD2, SMAD4, SMARCA4, SMARCB1, SMC1A, SMO, SOX9, SPOP, SPEN, SRC, STAG2, STAT3, STAT6, STK11, SUFU, TAP1, TAP2, TBX3, TCF7L2, TERT, TET2, TGFBR2, TNFAIP3, TNFRSF14, TOP1, TP53, TP63, TPMT, TPP2, TSC1, TSC2, U2AF1, USP8, USP9X, VHL, WT1, XPO1, XRCC2, XRCC3, YAP1, YES1, ZFHX3, ZMYM3, ZNF217, ZNF429, ZRSR2***Select Rearrangements (Inter and Intra-genic):***AKT1, AKT2, AKT3, ALK, AR, AXL, BRAF, BRCA1, CDKN2A, EGFR, ERBB2, ERBB4, ERG, ESR1, ETV1, ETV4, ETV5, FGFR1, FGFR2, FGFR3, MAP3K8, MET, MTAP, MYB, MYBL1, NOTCH1, NOTCH2, NOTCH3, NRG1, NTRK1, NTRK2, NTRK3, NUTM1, PIK3CA, PIK3CB, PPARG, PRKACA, PRKACB, RAF1, RARA, RELA, RET, ROS1, RSPO2, RSPO3, STAT6, TERT, TFE3, TFEB, YAP1*

**Supplementary discussion of genomic alterations**

CNV results were suboptimal, and no definite abnormalities were found. No gene fusions/ rearrangements were detected.

The AT-rich interaction domain 1A (*ARID1A*) gene, encodes a large nuclear protein belonging to the SWI/SNF chromatin remodeling complex, and is its most frequently mutated member [1]. In addition to epithelial malignancies, *ARID1A* mutations have also been reported in follicular lymphoma [2], as well as Burkitt lymphoma and lymphoplasmacytic lymphoma [3].

Splicing factor 3B subunit 1 (*SF3B1*) is the largest component of the SF3b protein complex, a component of RNA splicing machinery. Mutations in SF3B1 are most frequently reported in myelodysplastic syndrome, chronic myelomonocytic leukemia, acute myeloid leukemia, and chronic lymphocytic leukemia [4].

*TSC2* is a tumor suppressor gene which encodes for the growth inhibitory protein tuberin, part of the TSC protein complex. Mutations in *TSC2* have been associated with tuberous sclerosis and lymphangioleiomyomatosis [5]. It has rarely been reported in DLBCL [6]**.**

**Supplementary Table 1.** Clinical characteristics of patients with quadruple hit lymphoma.

| **Case #** | **Sex/ Age** | **Presentation** | **CNS Involvement** | **BM Involvement** | **LDH 110-225 IU/L** | **Ann Arbor Stage** | **IPI Score** |
| --- | --- | --- | --- | --- | --- | --- | --- |
| 1 [7] | M/72 | CNS Sx and generalized LAD | Positive | Negative | 582 | NA | NA |
| 2 [8] | F/81 | Bone marrow involved by B-cell lymphoma | NA | Positive | NA | NA | NA |
| 3 [8] | M/68 | Bone marrow involved by B-cell lymphoma | NA | Positive | NA | NA | NA |
| 4 [9] | F/51 | Diffuse lymphadenopathy | Negative | Negative | 522 | III | HI |
| 5 [10] | M/79 | CNS Sx, LAD, and skin infiltration | Positive | NA | 989 | III/IV | NA |
| 6 [11] | NA | NA | NA | NA | NA | NA | NA |
| 7 [12] | NA | NA | NA | NA | NA | NA | NA |
| 8 [12] | NA | NA | NA | NA | NA | NA | NA |
| 9 [13] | F/74 | Mediastinal mass | Negative | Negative | NA | II | NA |
| 10 [14] | M/76 | Cervical LAD | Negative | Negative | Normal | NA | H |
| 11 [Current case] | M/73 | Tonsillar mass and cervical LAD | Negative | Negative | Normal | I | L-I |

BCLUC: B-cell lymphoma unclassified, BM: bone marrow, CNS: Central nervous system, Sx: symptoms, F: female, HI: High intermediate, H: High, IPI: International Prognostic Index, LDH: lactate dehydrogenase, LAD: Lymphadenopathy, L-I: low to intermediate, M: male, NA: Not available, Sx: symptoms.

**Supplementary Table 2.** Histopathological features, diagnosis, ancillary studies, treatment, and outcome/overall survival of quadruple hit lymphomas.

| **Case #** | **Diagnosis** | **IHC** | **FISH/Cytogenetics** | **Treatment /**  **Response** | **Follow up/ Outcome** |
| --- | --- | --- | --- | --- | --- |
| 1 [7] | MCL Blastoid variant vs BCL UC | CD20+ CD10+ BCL1+ Bcl2+  CD3-  CD5-  CD23-  Bcl6-  Ki-67: 60% | t(3;14)(q27;q32) t(8;14)(q24;q32) t(11;22)(q13;q11) t(14;18)(q32;q21) | R-CHOP and intracranial MTX/ CR to LAD, PR to CNS | NA/ died of CNS disease |
| 2 [8] | DLBCL | NA | *BCL6R*/t(3;18)(q27;q21)  *MYC R*/t(8;14)(q24;q32)  *CCND1* R/t(11;14)(q13;q32)  *BCL2* R/t(14;18)(q32;q21) | Cyclophosphamide therapy | 9 days/ Death due to therapy related complications |
| 3 [8] | BL/mature B-ALL | NA | *BCL6R*/t(3;18)(q27;q21)  *MYC R*/t(8;14)(q24;q32)  *CCND1* R/t(11;14)(q13;q32)  *BCL2* R/t(14;18)(q32;q21) | NA | 6 days/ Death due to unknown causes |
| 4 [9] | DLBCL | CD10+ BCL2+ BCL6+ MUM1+ Ki67: 80% | *RCCND1*  *RMYC*  *RBCL2*  *RBCL6* | R-CHOP/ CR | 30 months/ NA |
| 5 [10] | DLBCL vs Pleomorphic MCL | BCL1+ BCL2+ BCL6+  SOX11- CD10-  Ki67: 80% | *RCCND1*  *RMYC*  *RBCL2*  *RBCL6* | Rituximab, Ifosfamide, Cytosine-arabinoside  Intrathecal MTX/ NA | 4 months/ Died of disease |
| 6 [11] | HGBCL with quadruple hits | NA | NA | Autologous BM transplantation | 8 months/ Died of relapse |
| 7 [12] | HGBCL | BCL1+ | *RCCND1*  *RMYC*  *RBCL2*  *RBCL6* | NA | NA |
| 8 [12] | HGBCL | BCL1+ | *RCCND1*  *RMYC*  *RBCL2*  *RBCL6* | NA | NA |
| 9 [13] | HGBCL with quadruple- hits | BCL1+ BCL2+ BCL6+ MUM1+ CD5- SOX11-CD10-  Ki67: 90% | *RCCND1*  *RMYC*  *RBCL2*  *RBCL6* | R-CHOP/ PR | 8 months/ Died of disease |
| 10 [14] | Quadrupl-hit pleomorphic MCL | CD20+ CD79a+ BCL6+ BCL1+  BCL2+ MYC+  CD5-  SOX11-  Ki67: 60% | *RCCND1*  *RMYC*  *RBCL2*  *RBCL6* | Lenalidomide in combination with R2-CHOP/ CR | 15 months NA |
| 11 [Current case] | HGBCL with BCL2, BCL6, MYC and CCND1 R | CD20+  BCL6+ BCL1+  BCL2+ cMYC-  CD5-  SOX11-  Ki67: 90% | *RCCND1*  *RMYC*  *RBCL2*  *RBCL6* | R-EPOCH/ CR | 20 months/ Alive |

ALL: acute lymphoblastic leukemia, BCLUC: B-cell lymphoma unclassified, BL: Burkitt Lymphoma, CNS: Central nervous system, CR: complete response, DLBCL: diffuse large B-cell lymphoma, FISH: fluorescence in situ hybridization, IHC: immunohistochemistry, HGBCL: high grade B-cell lymphoma, LAD = lymphadenopathy, R-CHOP:rituximab, cyclophosphamide, hydroxy daunomycin, vincristine sulfate, prednisone, MCL: mantle cell lymphoma, MTX: methotrexate, NA: not available, NGS: next-generation-sequencing, PR: partial response, R: rearrangement, R- EPOCH: rituximab, cyclophosphamide, hydroxy daunomycin, prednisone, etoposide,

**Supplementary Table 3.** Comparison of next-generation-sequence (NGS) results between current case and two previously reported cases of quadruple hit lymphoma

| **NGS** | | | | | | | | |
| --- | --- | --- | --- | --- | --- | --- | --- | --- |
| **Current Case** | | | [13] | | | [14] | | |
| Gene | AA change | VAF | Gene | AA change | VAF | Gene | AA change | VAF |
| ***ARID1A***  ***BCL2***  ***CCND3***  ***FANCA***  ***KMT2D***  ***MYC***  ***MYC***  ***MYC***  ***PIM1***  ***PIM1***  ***PIM1***  ***PIM1***  ***PXDNL***  ***SF3B1***  ***SOCS1***  ***TNFRSF14***  ***TSC2*** | p.Ser280AlafsTer111  -  p.Ser259Ala  p.Arg1011His  p.Arg2734Ter  p.Leu159Phe  p.Gln113His  p.Tyr89Asn  p.Tyr38Phe  p.Lys29Asn  p.Thr23Ile  p.Val96Leu  p.Thr453Ser  p.Lys666Asn  p.Ala16Thr  p.Gln158Ter  p.Gly20Ter | 11%  5%  42%  7%  43%  14%  13%  12%  9%  8%  7%  7%  8%  9%  7%  8%  8% | ***CDKN2A***  ***KRAS***  ***TP53***  ***TNFRSF14*** | p.R80*  p.G13D  p.F270S  p.T169fs*65 | 39%  35%  33%  32% | ***ARID1B***  ***BCL2***  ***BCL2***  ***BCL2***  ***CCND1***  ***CCND1***  ***CCND1***  ***FOXO1***  ***IL7R***  ***MGA***  ***PIM1*** ***SGK1***  ***SGK1*** ***SGK1*** | p.Gln454fs  p.Ala131Val  p.Arg146Lys  p.Val134Met  p.Ala121Ser  p.Glu162Ala  p.Thr184Ile  p.Thr24Ile  p.Phe213Leu  p.Gln1988*  p.Ser97Asn  p.Ala121Gly  p.Met385Ile  p.Pro190Ala | 9.05%  3.00%  28.00%  1.00%  19.00%  23.00%  3.00%  30.00%  21.00%  27.00%  22.00%  24.00%  27.00%  25.00% |

AA: amino acid, NGS: next-generation-sequence, VAF: variant allele frequency

**REFRENCES**

[1] J. Mullen, S. Kato, J. K. Sicklick, and R. Kurzrock, “Targeting ARID1A mutations in cancer,” *Cancer Treat Rev*, vol. 100, p. 102287, Nov. 2021, doi: 10.1016/j.ctrv.2021.102287.

[2] D. Bararia *et al.*, “ARID1A Is Recurrently and Significantly Mutated in Follicular Lymphoma (FL) and Impairs DNA Repair Efficiency,” *Blood*, vol. 124, no. 21, pp. 73–73, Dec. 2014, doi: 10.1182/blood.V124.21.73.73.

[3] E. Pavlidou and V. Balis, “Diagnostic significance and prognostic role of the ARID1A gene in cancer outcomes (Review),” *World Acad Sci J*, Jan. 2020, doi: 10.3892/wasj.2020.37.

[4] A. Samy, M. K. Ozdemir, and R. Alhajj, “Studying the connection between SF3B1 and four types of cancer by analyzing networks constructed based on published research,” *Sci Rep*, vol. 13, no. 1, p. 2704, Feb. 2023, doi: 10.1038/s41598-023-29777-5.

[5] J. Huang *et al.*, “Gene mutations in sporadic lymphangioleiomyomatosis and genotype–phenotype correlation analysis,” *BMC Pulm Med*, vol. 22, no. 1, p. 354, Sep. 2022, doi: 10.1186/s12890-022-02154-0.

[6] L. W. Cheung *et al.*, “Mutation Impact of Targeted Genes in Diffuse Large B-Cell Lymphoma Patients Treated with Ibrutinib,” *Blood*, vol. 126, no. 23, pp. 2642–2642, Dec. 2015, doi: 10.1182/blood.V126.23.2642.2642.

[7] K. Kawakami *et al.*, “Case of B-Cell Lymphoma with Rearrangement of the BCL1, BCL2, BCL6, and c-MYC Genes,” *Int J Hematol*, vol. 79, no. 5, pp. 474–479, Jun. 2004, doi: 10.1532/IJH97.03105.

[8] U. Bacher, T. Haferlach, T. Alpermann, W. Kern, S. Schnittger, and C. Haferlach, “Several lymphoma‐specific genetic events in parallel can be found in mature B‐cell neoplasms,” *Genes Chromosomes Cancer*, vol. 50, no. 1, pp. 43–50, Jan. 2011, doi: 10.1002/gcc.20831.

[9] M. Yoshida *et al.*, “Clinicopathological features of double‐hit <scp>B</scp> ‐cell lymphomas with *<scp>MYC</scp>* and *<scp>BCL</scp> 2* , *<scp>BCL</scp> 6* or *<scp>CCND</scp> 1* rearrangements,” *Pathol Int*, vol. 65, no. 10, pp. 519–527, Oct. 2015, doi: 10.1111/pin.12335.

[10] A. Ittel *et al.*, “Four genetic lymphoma-specific events (MYC, BCL2, BCL6 and CCND1) identified in a high grade B lymphoma case,” *Blood Cancer J*, vol. 5, no. 12, pp. e374–e374, Dec. 2015, doi: 10.1038/bcj.2015.99.

[11] J. Proulx *et al.*, “Quadruple Hit Lymphoma: A Rare Entity with Dismal Prognosis,” *Blood*, vol. 132, no. Supplement 1, pp. 5305–5305, Nov. 2018, doi: 10.1182/blood-2018-99-120147.

[12] A. Meloni-Ehrig *et al.*, “36. Quadruple-hit B-cell lymphoma with simultaneous BCL2, BCL6, CCND1, and MYC rearrangements: Two new cases,” *Cancer Genet*, vol. 244, p. 14, Jun. 2020, doi: 10.1016/j.cancergen.2020.04.040.

[13] J. Cheng *et al.*, “CCND1 Genomic Rearrangement as a Secondary Event in High Grade B-Cell Lymphoma,” *Hemasphere*, vol. 5, no. 1, p. e505, Jan. 2021, doi: 10.1097/HS9.0000000000000505.

[14] W. Liu *et al.*, “Quadruple‐hit pleomorphic mantle cell lymphoma with *MYC* , *BCL2* , *BCL6* , and *CCND1* gene rearrangements,” *Br J Haematol*, vol. 195, no. 4, pp. 634–637, Nov. 2021, doi: 10.1111/bjh.17729.
